# Protective Effects of Syringic Acid on Ischemia-Reperfusion Injury in Testicular Torsion: An Experimental Study in a Rat Model

**DOI:** 10.7759/cureus.42390

**Published:** 2023-07-24

**Authors:** Kubilay Sarikaya, Engin Kölükçü, Velid Unsal, Süleyman Özdemir

**Affiliations:** 1 Urology, Health Sciences University Ankara Etlik City Hospital, Ankara, TUR; 2 Urology, Tokat Gaziosmanpaşa University School of Medicine, Tokat, TUR; 3 Biochemistry, Faculty of Health Sciences and Central Research Laboratory, Mardin Artuklu University, Mardin, TUR; 4 Pathology, Faculty of Medicine, Gaziosmanpaşa University, Tokat, TUR

**Keywords:** oxidative damage, wistar rat, oxidant, reperfusion injury, testicular torsion

## Abstract

Aim: The purpose of this study was to assess the effectiveness of syringic acid in preventing ischemia-reperfusion injury following detorsion in a rat model of induced testicular torsion.

Material and methods: In our study, a total of 24 rats, eight in each group, were used. Group 1 served as the control group. Group 2 underwent testicular torsion and detorsion. Group 3 underwent the same procedures as Group 2, but also received 100 mg/kg syringic acid immediately following ischemia. Spectrophotometric analysis was performed on blood samples, and the activities of superoxide dismutase (SOD) and glutathione peroxidase (GSH-Px), as well as the values of malondialdehyde (MDA), were evaluated under direct microscopic examination of the testis to determine tissue injury. The architecture of the seminiferous tubules and spermatogenesis processes were graded using the Johnsen and Cosentino scoring systems.

Results: The mean value of MDA was higher in Group 2 compared to the other groups (p < 0.001). Group 3 demonstrated a decline in the concentrations of proinflammatory cytokines, such as tumor necrosis factor-alpha, interleukin-6, and interleukin-1 beta, as indicated by biochemical analysis of blood samples, when compared to Group 2 (p-values: 0.045, 0.001, and <0.001, respectively). In addition, the improvement in Johnsen and Cosentino scores was significantly higher in Group 3 compared to Group 2 (p = 0.028 and p = 0.001, respectively).

Conclusion: These findings suggest that syringic acid has a protective effect against testicular oxidative damage.

## Introduction

Acute scrotum, characterized by sudden scrotal pain, redness, and swelling, is considered a urological emergency. Testicular torsion, accounting for approximately 35% to 42% of acute scrotal conditions, is the cause of this uropathy [1]. The spermatic cord in this condition becomes twisted, interrupting testicular perfusion. Early intervention is crucial to avoid tissue damage due to ischemia. Rather than being used as a diagnostic tool, the standard approach for treating testicular torsion is to perform manual or surgical detorsion as an emergency procedure. According to the literature, manual or surgical detorsion within the first six hours is associated with high preservation rates of testicular tissue [2]. There is a wide variation in orchiectomy rates reported in the literature, with most series showing a range of 39% to 71% [3]. However, successfully treated cases of testicular torsion may experience long-term consequences, such as testicular atrophy, compromised semen quality, and infertility, related to ischemia-reperfusion injury [4]. In the pathophysiology of testicular parenchymal tissue injury, elevated levels of reactive oxygen species (ROS), increased intra-mitochondrial calcium levels, and cellular apoptosis are considered to be the main contributing factors [5]. To minimize the detrimental effects of ischemia-reperfusion injury, various medical treatment options in conjunction with manual or surgical detorsion are actively studied [5-7]. The elevation of ROS caused by testicular torsion/detorsion disrupts the normal functioning of vital cellular components, including intracellular genomic material, membrane lipids, and proteins. This interference ultimately leads to impaired sperm production and reduced sperm quality [8]. The role of free radical scavenging and antioxidant systems in protecting reproductive health is highly significant. Therefore, reducing oxidative stress levels plays a critical role in the development of treatment plans. By enhancing the activity of these systems and reducing oxidative stress, it is possible to mitigate the damaging effects of ROS and promote better reproductive health outcomes [9]. Syringic acid is a naturally occurring phenolic compound that is widely distributed in many plant species, including fruits, vegetables, and nuts. It has been extensively studied for its antioxidant properties, which make it a potential candidate for use in the food, pharmaceutical, and cosmetic industries [10]. The antioxidant properties of syringic acid are mainly attributed to its ability to scavenge free radicals and prevent oxidative damage to cells and tissues [11]. This is due to its chemical structure, which includes a hydroxyl group that can easily donate a hydrogen atom to neutralize free radicals. Additionally, syringic acid can also act as a metal chelator, helping to prevent the formation of toxic compounds that can cause oxidative damage [9]. Studies have shown that syringic acid has a number of health benefits, including reducing inflammation, preventing oxidative stress, and improving glucose tolerance [12]. It has also been shown to have neuroprotective effects and to be protective against cardiovascular disease, suggesting that it may have therapeutic potential for the treatment of a variety of health conditions [13]. The objective of the current study was to examine the potential protective effect of syringic acid in the treatment of ischemia-reperfusion injury following testicular torsion. As far as our knowledge extends, this study represents the first investigation in the English literature to administer syringic acid to rats with an induced testis torsion model.

## Materials and methods

This study investigated the effects of syringic acid on ischemia-reperfusion injury in rats. This study utilized a total of 24 male Wistar albino rats, aged 12 weeks, with an average weight of 325 g. The study was conducted in accordance with institutional guidelines and followed the principles outlined in the Guide for the Care and Use of Laboratory Animals of the National Research Council. Ethical approval for the study was obtained from the Tokat Gaziosmanpaşa University Animal Studies Ethical Committee (approval number: 2022-HAYDEK-17). The rats were housed in controlled conditions with a temperature range of 20-23°C and a 12-hour light/dark cycle. They were provided with standard pellets and water ad libitum to ensure their nutritional needs were met. The rats were randomly divided into three groups: Group 1 (control), Group 2 (ischemia-reperfusion), and Group 3 (treatment). Group 1 underwent only orchiectomy and no other treatments, while Group 2 underwent testicular torsion and detorsion with no medication administered. Group 3 received a single dose of 100 mg/kg of syringic acid via oral gavage immediately after ischemia with the syringic acid. Syringic acid was dissolved in dimethyl sulfoxide (DMSO) and diluted using 0.9% physiological saline solution just prior to administration. In Groups 2 and 3, ischemia-reperfusion injury was induced in the rat testes using the following method: the testis was pulled out through an inguinoscrotal incision, rotated 720 degrees in a clockwise direction, and then secured to the scrotum for a period of three hours using a 5.0 prolene suture. This procedure aimed to create a controlled model of testicular torsion to study the effects of ischemia and subsequent reperfusion. After the ischemic period, the testis was detorsioned and left in its normal anatomical position for another three hours to assess the reperfusion injury. In the final step of the procedure, orchiectomy was performed to obtain testis tissue for histopathological investigation, and blood samples were collected from the inferior vena cava for biochemical analysis. The surgical procedures were conducted under appropriate anesthesia and in sterile conditions. To achieve this, the rats were administered a combination of two anesthesia agents, i.e., xylazine hydrochloride (Rompun 2%, Bayer, Turkey) at a dose of 10 mg/kg via the intraperitoneal route and ketamine hydrochloride (Alfamine 10%, Ege Vet, Turkey) at a dose of 50 mg/kg administered via the intraperitoneal route. Xylazine hydrochloride acts as a sedative and muscle relaxant, while ketamine hydrochloride has dissociative anesthetic effects. These agents were used to ensure the rats' comfort and minimize pain during surgical procedures.

Biochemical evaluation

Measurement of Malondialdehyde Level

The study of lipid peroxidation was performed using the Esterbauer method, which measures the levels of malondialdehyde (MDA) in the sample. The method involves a reaction between MDA and thiobarbituric acid at 90-95°C, which creates a pink-colored chromogen. The intensity of the pink color is proportional to the amount of MDA present in the sample, and this can be used to quantify the level of lipid peroxidation. After the reaction between MDA and thiobarbituric acid, the samples were rapidly cooled and then spectrophotometrically read at 532 nm after 15 minutes. The level of MDA was then expressed as nmol/mL. This method, described by Esterbauer and Cheeseman in 1990, provides a way to measure the level of lipid peroxidation in a sample by quantifying the amount of MDA produced during the oxidation of lipids [14].

Measurement of Glutathione Peroxidase Activity

The activity of glutathione peroxidase (GSH-Px) was assessed using the method described by Paglia et al. [15]. This method measures the enzymatic ability of GSH-Px to facilitate the oxidation of reduced glutathione (GSH) to oxidized glutathione (GSSG) in the presence of hydrogen peroxide. GSH-Px acts as a scavenger of hydrogen peroxide, so the increase in the oxidation of nicotinamide adenine dinucleotide phosphate (NADPH) to nicotinamide adenine dinucleotide phosphate (NADP+) indicates the presence of GSH-Px and its ability to scavenge hydrogen peroxide. The activity of GSH-Px was determined by measuring the decrease in absorbance at 340 nm as NADPH was oxidized to NADP+. The activity of GSH-Px is expressed in units per liter (U/L). This method provides a way to measure the activity of GSH-Px in a sample and thus assess its role in protecting against oxidative stress.

Measurement of Superoxide Dismutase Activity

The activity of the superoxide dismutase (SOD) enzyme was assessed by modifying the method described by Sun et al. [16]. This method utilizes the reduction of nitroblue tetrazolium (NBT) by the superoxide generated from the xanthine-xanthine oxidase system. SOD acts as a scavenger of superoxide, so a decrease in NBT reduction indicates the presence of SOD and its ability to scavenge superoxide. The activity of SOD is expressed as U/L. This method provides a way to measure the activity of SOD in a sample and thus assess its role in protecting against oxidative stress.

Measurement of Tumor Necrosis Factor Alpha, Interleukin-1 Beta, Interleukin-6 Levels

Tumor necrosis factor alpha (TNF-alpha), interleukin-1 beta (IL-1 beta), and interleukin-6 (IL-6) levels were quantified using ELISA kits on the Thermo Scientific Multiskan FC Microplate device (Thermo Fisher Scientific, Waltham, MA), following the manufacturer's instructions. The ELISA kits were sourced from BioAssay Technology Laboratory, and their % CV (coefficient of variation) values were below 10, indicating reliable assay reproducibility.

Histopathological evaluation

The testes taken from the rats were preserved in a 10% formalin solution buffered with a buffer for a duration of two days. After being trimmed and processed, the samples were cut into 4-micrometer sections using a microtome and then stained with hematoxylin and eosin dyes. The tissue slides were examined using an upright light microscope (Nikon Eclipse E600, Tokyo, Japan). The pathologist was blinded to the groups. The structure of the seminiferous tubules, the processes of spermatogenesis, and the maturity of germ cells were evaluated using the Johnsen and Cosentino scoring systems. The Johnsen scoring criteria, which range from 1 to 10, were used to evaluate the samples as follows: a score of 1 indicates fibrosis, with only Leydig and peritubular myoid cells present and germ and Sertoli cells absent; 2 indicates Sertoli cells are present but do not involve germ cells; 3 indicates only spermatogonia are present; 4 indicates spermatogonia and few spermatocytes are present; 5 indicates no spermatozoa or spermatids are present, but many spermatocytes are present; 6 indicates few early spermatids are present; 7 indicates spermatogonia, spermatocytes, and early spermatids are present but no spermatozoa and late spermatids; 8 indicates only a few spermatozoa are present; 9 indicates spermatogonia, spermatocytes, spermatids, and spermatozoa are present but disorganized; 10 indicates normal spermiogenesis [16]. The injury to the testicular parenchyma was evaluated using the Cosentino scoring system. Coagulation necrosis was rated with 1 for normal and 4 for extensive necrosis [17]. The histopathological examination was conducted by a single pathologist to ensure consistency.

Statistical analysis

All statistical analyses were performed using SPSS 22.0 software (IBM Corp., Armonk, NY) for Windows. P < 0.05 was considered statistically significant in all analyses. The Shapiro-Wilk test was used to assess the distribution. For analysis, the t-test, one-way analysis of variance (ANOVA) test, and post hoc Bonferroni analysis were used for parametric variables, and the Mann-Whitney U test and Kruskal-Wallis tests were used for nonparametric variables. Data are presented as a median and interquartile range for nonparametric variables and mean ± standard deviation for parametric variables.

## Results

In Group 1, the mean levels of TNF-alpha, IL-6, and median IL-1 beta were measured as 486.5 ± 94.2 ng/ml, 12.1 ± 3.3 ng/ml, and 598 (508-721) pg/ml, respectively. Group 2 exhibited significantly higher levels of pro-inflammatory cytokines, including IL-1 beta, IL-6, and TNF-alpha (p < 0.001, p < 0.001, and p < 0.001, respectively) when compared to Group 1. In Group 3, the mean levels of TNF-alpha and IL-6 and the median value of IL-1 beta were measured as 591.8 ± 85.8 ng/ml, 12.7 ± 2.6 ng/ml, and 598 (501-759) pg/ml, and they were found to be significantly lower than those in Group 2 (p = 0.042, p = 0.001, and p < 0.001, respectively) (Table 1).

**Table 1:** Comparison between TNF-alpha, IL-6, and IL-1 beta values in three rat groups TNF-alpha: tumor necrosis factor alpha; IL-6: interleukin-6; IL-1 beta: interleukin 1-beta.

	Groups	Mean ± SD	P-value	Post-hoc p-values
TNF-alpha	1	486.5 ± 94.2	<0.001	1-2: <0.001
	2	690.2 ± 37.5		1-3: 0.036
	3	591.8 ± 85.8		2-3: 0.042
IL-6	1	12.1 ± 3.3	<0.001	1-2: <0.001
	2	19.3 ± 2.7		1-3: 1
	3	12.7 ± 2.6		2-3: 0.001
		Median (min-max)		
IL-1 beta	1	598 (508-721)	<0.001	1-2: <0.001
	2	799 (780-998)		1-3: 0.878
	3	598 (501-759)		2-3: <0.001

The activities of serum antioxidant enzymes SOD and GSH-Px were found to be significantly lower in Group 2 compared to Group 1 (p = 0.001 and p = 0.002, respectively). Although there was an increase in SOD and GSH-Px activities in Group 3 compared to Group 2, no statistically significant difference was found between the groups (35.1 ± 6.4 vs. 29.7 ± 6.1 and 5.4 ± 1.4 vs. 4.3 ± 1.5; p = 0.306 and p = 0521, respectively). Additionally, the levels of MDA, a marker of oxidative stress, were found to be significantly higher in Group 2 compared to Group 1 (p < 0.001). When Group 3 was analyzed, a decrease in MDA levels was noted compared to the increase in Group 2 (p = 0.001) (Table 2).

**Table 2:** Comparison between GSH-Px, SOD, and MDA values in three rat groups SOD: superoxide dismutase; GSH-Px: glutathione peroxidase; MDA: malondialdehyde.

	Groups	Mean ± SD	P-value	Post-hoc p-values
SOD (U/L)	1	43.9 ± 6.2	0.001	1-2: 0.001
	2	27.9 ± 6.1		1-3: 0.033
	3	35.1 ± 6.4		2-3: 0.306
GSH-Px (U/L)	1	7.5 ± 1.7	0.002	1-2: 0.002
	2	4.3 ± 1.5		1-3: 0.044
	3	5.4 ± 1.4		2-3: 0.521
MDA (nmol/ml)	1	6.1 ± 1.0	<0.001	1-2: <0.001
	2	10.3 ± 2.1		1-3: 0.482
	3	7.1 ± 0.9		2-3: 0.001

The Johnsen and Cosentino scoring after ischemia-reperfusion injury revealed negative alterations in Group 2 compared to Group 1 (p < 0.001 and p = 0.001, respectively). However, when the same scores were analyzed in Group 3, positive changes were observed compared to Group 2 (p < 0.028 and p = 0.001, respectively) (Table 3).

**Table 3:** Comparison of Johnsen and Cosentino scores in three rat groups

	Groups	Median (min-max)	P-value	Post-hoc p-values
Johnsen	1	10 (9-10)	<0.001	1-2: <0.001
	2	8 (7-9)		1-3: 0.001
	3	9 (8-9)		2-3: 0.028
Cosentino	1	1 (1-2)	<0.001	1-2: 0.001
	2	3 (2-3)		1-3: 0.442
	3	2 (1-2)		2-3: 0.001

**Figure 1:**
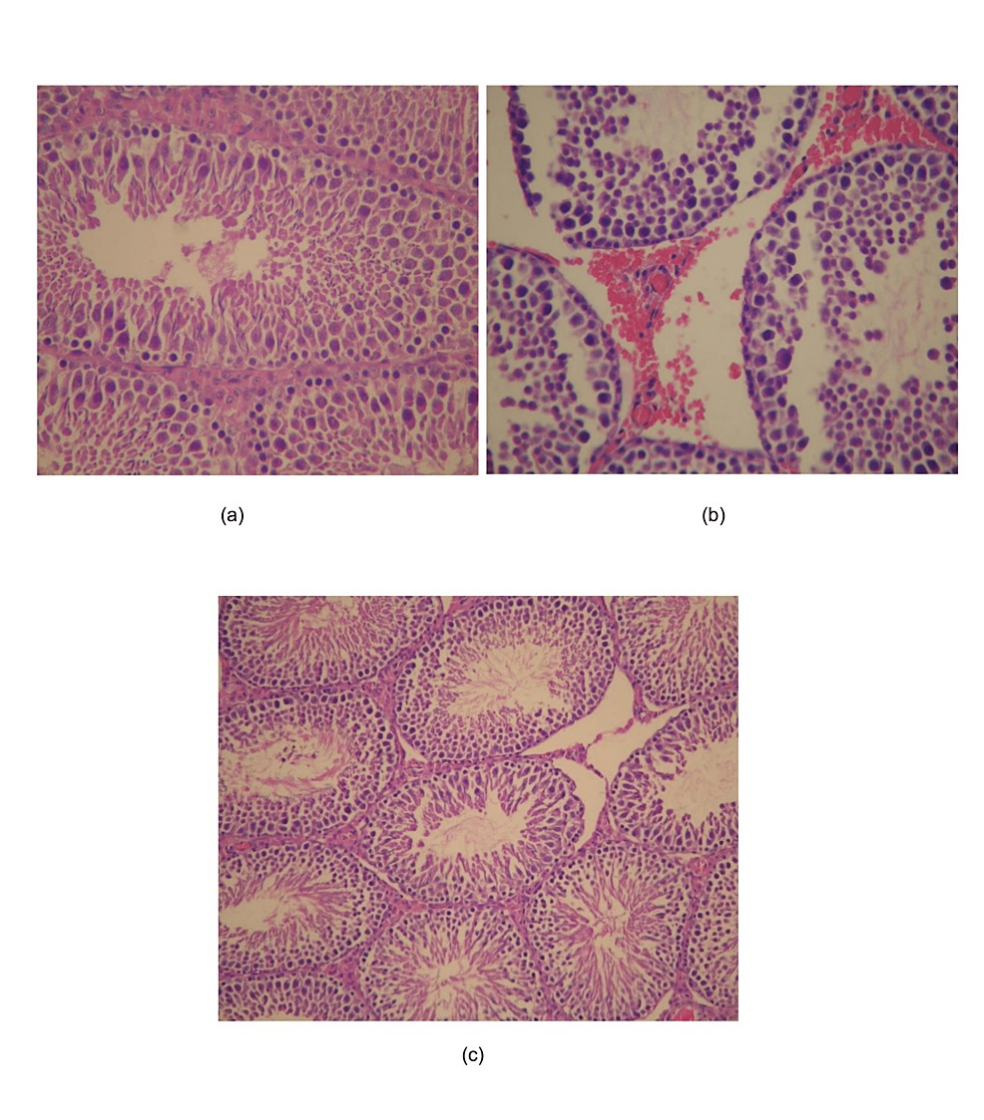
Johnsen's scores of the groups (a) Group 1: Testicular tissue with normal spermatogenesis was observed and Johnsen's score was 10. Magnification is ×400. (b) Group 2: In the injured testicular tissue, spermiogenesis was observed, and spermatogonia and spermatocytes were present. However, late spermatids and spermatozoa were not evident in the affected tubules. According to Johnsen's scoring system, a score of 7 was assigned. Magnification is ×400. (c) Group 3: Testicular tissue with disorganized spermatogonia, spermatocytes, spermatids, and spermatozoa. Johnsen's score was 9. Magnification is ×200.

**Figure 2:**
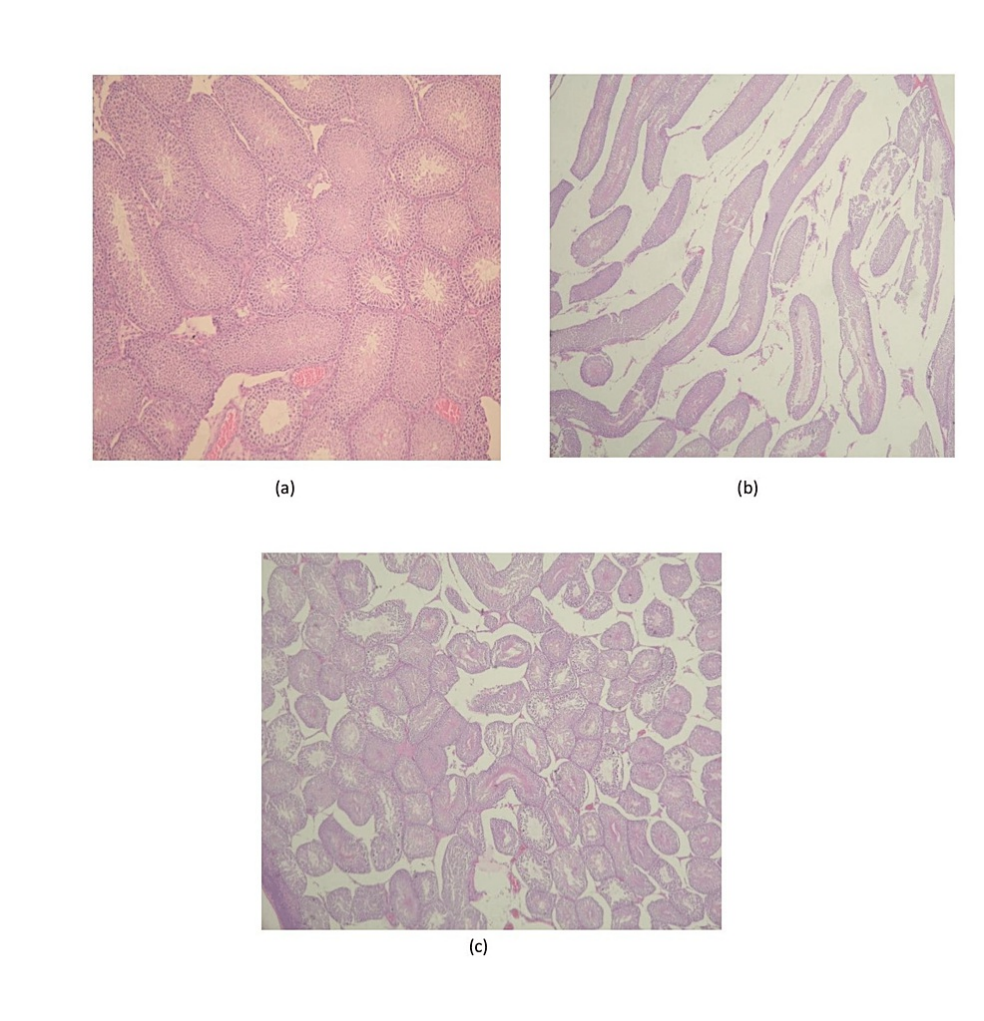
Evaluation of tubule architecture and injury in three groups (a) Group 1: Normal seminiferous tubule image without any injury. Magnification is ×200. (b) Group 2: Seminiferous tubule architecture was distorted widely in some tubules. Magnification is ×200. (c) Group 3: Focal cellular injury and alterations were detected in some seminiferous tubules. Magnification is ×200.

The tubule architecture and spermiogenesis evaluation are depicted in Figures 1, 2.

## Discussion

Testicular torsion is an urgent urological condition that needs immediate intervention. This condition has an annual frequency of occurrence of one in 4000 males aged between one and 25 years [18]. Timely diagnosis and intervention play a critical role in maintaining both testicular function and fertility since testicular ischemic damage of a significant proportion can occur after four to eight hours. The salvage rates of the testicles, as reported, range from 90% to 100% when surgical exploration is carried out within six hours of the onset of symptoms. The rates decrease to 50% when symptoms persist for more than 12 hours, and typically fall below 10% when the duration of symptoms is 24 hours or more [19]. There is a wide variation in orchiectomy rates reported in the literature, with most series showing a range of 39% to 71% [20]. Surgical intervention is the primary approach to address testicular torsion, which entails the reversal of testicular torsion and the reinstatement of blood flow to the testis. Regardless of the viability of the affected testis, contralateral orchiopexy ought to be carried out [21]. If immediate surgical intervention is not possible or if preparations for surgical exploration are in progress, one may consider attempting manual detorsion. Although manual detorsion is another conservative approach, it is never an alternative to surgical detorsion [22]. Mammalian testes possess a heightened susceptibility to oxidative stress owing to the elevated levels of polyunsaturated fatty acids found in their cell membranes, coupled with their constant cellular division and spermatogenesis [3,7,8]. Testicular torsion has a significant impact on spermatogenesis. Torsion-induced reduction results in DNA harm, inhibition of protein synthesis, and interruption of spermatogonia proliferation, all of which impede spermatogenesis. Testicular ischemia-reperfusion injury disturbs spermatogenesis following testicular torsion, and the inflicted damage may persist while causing short-term suppression of testosterone secretion [23]. This phenomenon has the potential to render the testes aspermatogenic.

Hence, an assortment of antioxidant pharmaceuticals, enzymes, and chemical compounds may be employed to enhance the functions of antioxidant enzymes, impede oxidative stress, and forestall ischemia/reperfusion harm, such as sildenafil citrate, α-lipoic acid, colchicine, and *Ginkgo biloba* [24]. In this context, syringic acid (4‐hydroxy‐2,3‐dimethoxybenzoic acid) is a naturally occurring phenolic acid that is acknowledged as an innate antioxidant capable of extinguishing free radicals [25]. This feature can be ascribed to its capacity for electron donation as well as its steady phenoxy radical intermediate [26]. The radical suppression capability of syringic acid has been linked to its inhibitory impact on lipid peroxidation and GSH reduction in the hepatic, renal, and neuronal tissues of rats with diabetes. Rashedinia et al. examined the effect of syringic acid on diabetic nephropathy and mitochondrial biogenesis [27]. According to their study, rats were orally given syringic acid at doses of 25, 50, and 100 mg/kg/day for a duration of six weeks. The administration of syringic acid led to a significant decrease in blood glucose levels and alkaline phosphatase (ALP) levels in the rats. Additionally, syringic acid administration in the diabetic group increased kidney GSH content and restored elevated renal catalase and SOD activities to normal levels.

Additionally, in diabetic rats, the administration of syringic acid significantly decreased the renal TBARS (thiobarbituric acid reactive substances) level, which had initially increased. In diabetic rats, the administration of syringic acid resulted in an upregulation of renal mRNA expression of PGC-1α (peroxisome proliferator-activated receptor-gamma coactivator) and NRF-1 (nuclear respiratory factor 1). Additionally, it increased the mitochondrial DNA (mtDNA)/nuclear DNA (nDNA) ratio, which indicates mtDNA content relative to nDNA, whereas these values were reduced in the diabetic group that did not receive the treatment. Thus, the authors concluded that syringic acid modulates renal antioxidant defense mechanisms. In another study, Sabahi et al. conducted a study to assess the protective effect of syringic acid against diabetes-induced cardiac injury in experimental rats [28]. In their study, the rats were divided into control and streptozotocin-induced diabetic groups, with the latter further subdivided into diabetic controls and three test groups (syringic acid at 25, 50, and 100 mg/kg). The non-diabetic group was administered 100 mg/kg of syringic acid for six weeks. The researchers reported that treatment with syringic acid (50 and 100 mg/kg) significantly reduced (p < 0.01 and p < 0.001, respectively) levels of TBARS in the hearts of diabetic rats. However, treatment with syringic acid 100 mg/kg significantly decreased the activities of chloramphenicol acetyltransferase (CAT) and SOD in diabetic rats (p < 0.001 and p < 0.05, respectively). At the conclusion of this study, it was indicated that syringic acid can reduce the effects of diabetic cardiomyopathy by improving the levels of creatine kinase-myocardial band (CK-MB) and lactate dehydrogenase (LDH), reducing lipid peroxidation and protein carbonylation in the heart. In another similar study, Han et al. examined the effects of syringic acid on cardiac hypertrophy and fibrosis in isoproterenol-treated mice and cells [29]. In the results of their study, it was stated that syringic acid was found to mitigate isoproterenol-induced upregulation of Ereg, Myc, and Ngfr. Additionally, Ereg knockdown mitigated the upregulation of Nppb and Fn1 and the enhancement of cell size. The mechanism of action was that syringic acid downregulated Ereg, resulting in the alleviation of cardiac hypertrophy and fibrosis. The results of the study suggest that syringic acid has the potential to be used as a therapeutic agent for treating cardiac hypertrophy and fibrosis.

In our study, we found that Cosentino and Johnsen scoring systems, which assess the morphology of seminiferous tubules, germ cell morphology, and spermatogenesis processes, demonstrated a negative change in response to ischemia-reperfusion injury. However, a significant improvement was observed in Johnsen and Cosentino scores in Group 3, where we gave 100 mg/kg syringic acid following testicular ischemia, compared to Group 2, which was not treated in our study. Significant improvement in Cosentino and Johnsen's scores observed in Group 3 compared to Group 2 supports the idea that syringic acid has a protective effect against oxidative damage. Furthermore, we observed an increase in the levels of MDA, which is an end product of lipid peroxidation, in rats with induced ischemia-reperfusion injury. In our study, it was determined that the MDA level decreased significantly in Group 3 with the application of syringic acid (100 mg/kg) following testicular ischemia. Additionally, there was a decrease in the levels of SOD and GSH-Px, which are important antioxidant enzymes in organisms, indicating a decline in antioxidant enzyme capacity in our study. Although there was no statistically significant difference in our study, it was determined that there was less decrease in SOD and GSH-Px values in Group 3 compared to Group 2. This may be due to the small number of rats included in our study, but it suggests that this difference may become more significant in studies involving more rats. It has been shown that there is an increase in proinflammatory cytokines such as TNF-alpha, IL-6, and IL-1 beta in the serum due to ischemia-reperfusion injury in several studies [30]. Consistent with the literature data, in our study, a significant increase in serum TNF-alpha, IL-6, and IL-1 beta levels was detected secondary to testicular ischemia. However, serum TNF-alpha, IL-6, and IL-1 beta levels were found to be significantly lower in Group 3, where syringic acid (100 mg/kg) was administered following ischemia, compared to Group 2.

Our study provided evidence that syringic acid has a protective effect against ischemia-reperfusion injury in testicular tissue. Additionally, the present study has yielded promising data regarding the use of syringic acid in routine urology practice to mitigate the effects on the reproductive system in cases of testicular torsion. However, to further validate our findings, randomized prospective clinical trials are needed. These trials will provide additional support for the efficacy of syringic acid in treating testicular torsion. Furthermore, more comprehensive studies should be conducted to determine the appropriate dosage range and optimal timing for administering syringic acid in different timeframes of testicular torsion. We believe that future studies should focus on revealing the potential side effects of syringic acid when used for its protective effect against testicular damage. Additionally, it would be important to develop appropriate algorithms to address any potential complications associated with the administration of the drug. This will contribute to a better understanding of the safety profile and facilitate the optimal use of syringic acid in clinical settings.

Limitations

The main limitation of our study is that we focused solely on the early effects of syringic acid on testicular torsion, administering only a single dose. Consequently, we were unable to investigate the long-term response of testicular tissue to this pharmacological agent. Future studies should consider assessing the prolonged effects and optimal dosing regimen of syringic acid for testicular torsion. In addition to the aforementioned limitation, it is important to acknowledge other limitations of our study. First, we were unable to demonstrate the effects of syringic acid on testicular tissue in healthy rats, which would have provided valuable comparative data. Furthermore, we did not evaluate hormone profiles, which could have provided additional insights into the mechanisms of action. Lastly, the absence of immunohistochemical analyses in our pathological examinations limited our ability to investigate specific cellular markers and molecular pathways. These limitations highlight areas for improvement and future research directions in studying the effects of syringic acid on testicular tissue.

## Conclusions

In our study, we found that the administration of syringic acid attenuated tissue damage caused by ischemia-reperfusion. Specifically, we observed an increase in the serum levels of antioxidants, such as SOD and GSH-Px, while the levels of MDA, a marker of lipid peroxidation, decreased. Furthermore, in rats treated with syringic acid, we observed improvements in seminiferous tubule morphology, spermatogenesis processes, and scores according to both the Johnsen and Cosentino scoring systems, indicating enhanced germ cell maturation. According to the findings from this experimental investigation, the utilization of syringic acid presents itself as a viable alternative therapeutic approach to alleviate the ischemia-reperfusion injury following detorsion interventions in individuals afflicted with testicular torsion. To gain a better understanding of the molecular mechanism underlying the efficacy of syringic acid, it is important to conduct prospective, randomized, and controlled clinical studies in the future. These studies will provide more robust evidence and further validate the findings obtained in our study.
